# Health status, sexual activity and satisfaction among older people in Britain: A mixed methods study

**DOI:** 10.1371/journal.pone.0213835

**Published:** 2019-03-27

**Authors:** Bob Erens, Kirstin R. Mitchell, Lorna Gibson, Jessica Datta, Ruth Lewis, Nigel Field, Kaye Wellings

**Affiliations:** 1 Department of Health Services Research and Policy, London School of Hygiene & Tropical Medicine, London, United Kingdom; 2 MRC/CSO Social and Public Health Sciences Unit, Institute of Health and Wellbeing, University of Glasgow, Glasgow, United Kingdom; 3 Department of Public Health, Environments and Society, London School of Hygiene & Tropical Medicine, London, United Kingdom; 4 Institute for Global Health, University College London, London, United Kingdom; University of Malaya, MALAYSIA

## Abstract

**Background:**

Men and women are increasingly likely to stay sexually active into later life, but research shows that sexual activity and satisfaction decrease with increasing age. Ill health and medical treatments may affect sexual activity but there is little research on why some older people with a health problem affecting their sexual activity are satisfied with their sex life, and others are not.

**Methods:**

A mixed method study integrating data and analyses from the third British National Survey of Sexual Attitudes and Lifestyles (n = 3343 aged 55–74 years) and from follow-up in-depth interviews with 23 survey participants who reported having a health condition or taking medication affecting their sex life.

**Results:**

Overall, 26.9% of men and 17.1% of women aged 55–74 reported having a health condition that affected their sex life. Among this group, women were less likely than men to be sexually active in the previous 6 months (54.3% vs 62.0%) but just as likely to be satisfied with their sex life (41.9% vs 42.1%). In follow-up interviews, participants sometimes struggled to tease out the effects of ill health from those of advancing age. Where effects of ill health were identified, they tended to operate through the inclination and capacity to be sexually active, the practical possibilities for doing so and the limits placed on forms of sexual expression. In close relationships partners worked to establish compensatory mechanisms, but in less close relationships ill health provided an excuse to stop sex or deterred attempts to resolve difficulties. Most fundamentally, ill health may influence whether individuals have a partner with whom to have sex.

**Conclusion:**

The data show complex interactions between health, lifestyle and relationship factors that affect sexual activity/satisfaction. When dealing with sexual problems in older people, practitioners need to take account of individual lifestyle, needs and preferences.

## Introduction

Attitudes towards, and experience of, sexual activity in later life have changed in recent decades. Many men and women remain sexually active well into later life [1–5], and the proportion who do so is growing. Surveys show an increase over time in the proportion of 70 year olds who are sexually active, who see sexuality as a positive force in life and express satisfaction with their sex lives [6]. Several trends help to explain this. Men and women today live longer and reach older age in better health [7]; and–perhaps most notably—social attitudes towards sex in later life have relaxed. Today, sexual expression is increasingly recognised as important throughout the life course, in maintaining relationships, promoting self-esteem and contributing to health and well-being [8–10].

There is, nevertheless, evidence that sexual expression changes with increasing age. Studies have shown a decline in sexual function with advancing years [2, 5, 11, 12] and, more equivocally, a lessening of desire among women [6, 13]. Age-related decreases in sexual activity and satisfaction have been shown in a large number of studies for both men and women [1, 3, 5, 11, 13–16].

The decline in sexual activity and satisfaction can be attributed to various factors including the loss of a long-term sexual partner; deterioration in a continuing relationship; changes in hormonal status; and alteration in physical appearance impacting on self-esteem and response [1, 2, 6, 16]. A key factor is the impact of declining health (and medications for ill-health) on sexual function [1, 11, 15].

The list of conditions with the potential to impact on sexual activity and satisfaction is long and includes diabetes, cardiovascular disease, prostate cancer, chronic airways disease, musculo-skeletal disorders and neurological impairment, and some cancers [1, 2, 5, 13, 15, 17–20]. Depression has also been shown to be associated with poorer sexual function, although cause and effect are not easily established [9, 12, 16, 21].

The growing body of literature has been partly stimulated by the advent of phosphodiesterase-5 (PDE-5) inhibitors (i.e., sildenafil, brand name Viagra). With rare exceptions [22, 23], research on health and sexual dysfunction reflects a predominantly biomedical perspective, potentially overlooking key aspects of function such as the relational context and patient appraisal of whether a problem exists [24]. Many studies are clinically based, proximate to the recent experience of ill health, and document physician-based remedies rather than patient-centred solutions. There is little research on how older people themselves see their health as impacting on their sexual expression and how they respond to this. Empirical evidence is also lacking on why some older people who report having a health problem affecting their sexual activity are dissatisfied with their sex life, while others are not, or how the sexual response is influenced by relationship status and quality. As a result, there is little to guide practitioners in helping to improve sexual satisfaction and experience among older men and women with health concerns.

This study had two aims: to explore how older people see their health status as having influenced their sexual activity and satisfaction; and secondly, to further understanding of how they respond and deal with the consequences.

Natsal-3 was approved by the Oxford Research Ethics Committee A [Ref: 10/H0604/27] The qualitative data collection was approved by the same committee [Ref: 10/H0604/10].

## Materials and methods

We carried out a mixed method study that integrated data from the third British National Survey of Sexual Attitudes and Lifestyles (Natsal-3) with follow up in-depth interviews drawn from a sub-sample of participants aged 55–74 years who reported in the survey having a health condition which affected their sex life in the last year. We describe the prevalence of sexual activity and satisfaction among this group, and draw on in-depth interviews to explore ways in which health can impact on sexual activity and satisfaction.

In combining qualitative and quantitative data we sought to exploit synergies between different approaches to examining the same phenomena. The qualitative data were used to illuminate associations found in the survey data and findings from the qualitative research were, in turn, used to shape analysis of the survey data.

### Quantitative component

Natsal-3 is a probability sample survey of men and women aged 16–74 years living in private households in Britain. Overall, 15,162 adults were interviewed, of whom 3,343 were aged between 55–74 years at interview. The response rate for Natsal-3 was 57.7% and the co-operation rate was 65.8%. Full details of Natsal-3 methods have been reported elsewhere [25, 26].

The two outcomes were ‘sexual activity’, defined as having had vaginal, oral or anal intercourse with one or more opposite-sex or same-sex partners in the past six months, and ‘sexual satisfaction’, measured using a Likert scale probing agreement with the statement, “*I feel satisfied with my sex life*” (in the last year), with ‘agree’ and ‘agree strongly’ responses combined to categorise satisfaction (and neither agree nor disagree, disagree, and disagree strongly categorised as ‘other’).

Variables included in the analysis were age at interview; relationship status; economic status; self-reported health (a scale from ‘excellent’ to ‘bad/very bad’); experience of longstanding illness or disability; and experience of depressive symptoms in the past two weeks using the Patient Health Questionnaire (PHQ-2). Body mass index (BMI) was calculated from self-reported height and weight, and mobility was assessed by asking about ease of walking up a flight of stairs. Other variables included: whether participants sought help or advice about their sex life in the past year; ever used medication to assist sexual performance; level of agreement with the statement: “*It’s natural for people to want less sex as they get older*”; and whether they found it easy or difficult to talk to their regular partner about sex. For those who had been in a relationship for the entire previous year, participants were asked how happy they were in their relationship on a scale ranging from 1 (happy) to 7 (unhappy), with responses 1 and 2 coded as ‘happy’. Additional variables included whether participants had vaginal intercourse, oral sex or genital contact without intercourse in the last six months and satisfaction with the current amount of sexual activity.

The prevalence of having had a health condition or disability, or taken any medication, in the last year which affected sexual activity or enjoyment was estimated among Natsal-3 participants aged 55–74 years. Among the sub-group of participants reporting health conditions, disability or medication affecting their sex life, we estimated sexual activity in the last six months, and satisfaction with current sex life, in relation to selected lifestyle, health-related and relationship factors. After analysing half of the in-depth interviews, statistical analyses were re-run adding variables which emerged as important in participants’ depth accounts, including happiness with the relationship with their (most recent) partner and the repertoire of sexual practices engaged in during the previous year. Regression analysis was used to adjust for age and relationship status.

Analyses were carried out using the complex survey functions of Stata (version 14) and were weighted to adjust for the unequal probabilities of selection and for differential non-response. We report prevalence estimates and 95% confidence intervals (CIs) separately for men and women.

### Qualitative component

Participants eligible for the qualitative study were the 388 men and 281 women aged 55–74 years who reported in Natsal-3 having had, in the last year, a health condition or disability, or taken any medication, affecting their sex life. Participants were asked whether they would be willing to be contacted again about taking part in another interview and 80% of the eligible group agreed. A sample was drawn, guided by: the recency with which Natsal-3 interviews had been conducted; the need for roughly equal numbers of men and women; and a geographical spread across Britain (reflecting the quantitative survey). Governing the final sample size was the need to achieve sufficient variation in individual experience to explore the issues of interest and ensure saturation of themes. Letters were sent to participants inviting them to take part in a further interview, followed by a phone call from a researcher to explain the purpose of the interview, check on willingness to take part and arrange interviews.

Participants gave signed consent and were provided with an information sheet and a list of agencies from which they could seek advice on topics raised. In-depth interviews with 11 men and 12 women were carried out face-to-face in participants’ homes, recorded with their agreement and transcribed. The sex, age and relationship status of the participants are shown in Table 1.

**Table 1:** Characteristics of in-depth interview participants.

Participant	Gender	Age	Relationship status
M1	Male	75	Married
M2	Male	61	Married
M3	Male	69	Married
M4	Male	62	Married
M5	Male	58	Single
M6	Male	63	Separated
M7	Male	64	Married
M8	Male	64	Married
M9	Male	62	Widowed, new partner
M10	Male	70	Single
M11	Male	71	Married
W1	Female	71	Married
W2	Female	69	Married
W3	Female	67	Separated
W4	Female	71	Married
W5	Female	69	Married
W6	Female	59	Cohabiting
W7	Female	68	Non-cohabiting regular partner
W8	Female	74	Married
W9	Female	64	Married
W10	Female	65	Married
W11	Female	59	Widowed
W12	Female	59	Married

The topic guide, refined during fieldwork, explored: perceptions of the relationship between health status and sexual activity and enjoyment; how health issues affected sexual activity; the relationship context; and action taken by participants in response to health-related sexual problems.

We undertook a thematic analysis drawing on principles of grounded theory (e.g open coding; constant comparison) [27]. Key themes emerged from close reading of transcripts, and open coding of transcript portions, focusing on excerpts that illuminated the relationship between ill-health and sexual frequency and satisfaction. The coding frame emerging from this exploratory phase included higher order (e.g. relationship context) and lower order (e.g. partner response to difficulties) themes, some of which were descriptive (e.g. beliefs about ageing) and some conceptual (e.g. sexual scripts). Grouping of higher order and lower order themes was guided by the need to explore the nature of the association between health and sexual activity and enjoyment, the ways in which participants saw health conditions impacting on their sexual activity and enjoyment, and their responses to this. Coding and analysis of the data was undertaken in NVivo (KM) and manually (KW). The analysis was refined with the aid of text searches in NVivo, identification of ‘deviant’ cases, dialogue between authors, and by continual back and forth between raw data and the emerging synthesis and summary of the data.

## Results

### Reporting a health condition affecting sexual activity or satisfaction, among all participants aged 55 to 74

Among Natsal-3 participants aged 55–74 years, roughly one in four men (26.9%) and one in six women (17.1%) reported having a health condition, or taking medication, that had affected their sexual activity or satisfaction in the previous 12 months (shortened to ‘having a health condition’ for this paper) (Table 2). The prevalence was considerably higher among women with a cohabiting or steady partner compared with those without, a difference which was less marked among men. Those with lower self-rated health and mobility, with higher BMI (men only), with more self-reported chronic conditions, or with reported longstanding illness or disability were more likely to report having a health condition affecting their sexual activity or satisfaction.

**Table 2:** Reporting a health condition/medication that affected sex life within last year in relation to demographic and health characteristics, by sex.

	Men			Women		
*Aged 55–74*	Bases unweighted, weighted	%	(95% CI)	Bases unweighted, weighted	%	(95% CI)
**All**	1283, 1857	26.9	(24.4–29.6)	1767, 2023	17.1	(15.1–19.2)
**Age:**						
55–64	698, 1095	25.4	(22.1–28.9)	975, 1175	18.8	(16.2–21.7)
65–74	585, 762	29.2	(25.2–33.5)	792, 848	14.8	(12.1–17.9)
**Relationship status**						
Living with partner/steady relationship	932, 1542	27.8	(24.8–31.0)	1088, 1463	20.5	(17.9–23.3)
Not in steady relationship	350, 314	22.6	(18.2–27.9)	675, 557	8.3	(6.2–11.0)
**Self-reported general health**						
Very good	319, 468	13.2	(9.7–17.8)	518, 607	8.8	(6.3–12.1)
Good	547, 797	20.9	(17.5–24.7)	698, 811	12.1	(9.6–15.1)
Fair	304, 434	40.6	(34.7–46.7)	399, 431	25.9	(21.3–31.2)
Bad/very bad	112, 155	60.0	(49.2–69.8)	152, 173	47.6	(38.7–56.7)
**Longstanding illness/disability**						
None	566, 832	13.3	(10.4–16.8)	812, 957	8.8	(6.7–11.4)
Not limiting	317, 480	26.9	(22.2–32.2)	406, 460	12.1	(9.1–16.1)
Limiting	399, 544	47.9	(42.3–53.4)	549, 607	34.0	(29.5–38.7)
**Body Mass Index (BMI)**						
Underweight/normal <25 kg/m^2^	416, 582	21.5	(17.5–26.0)	656, 751	16.7	(13.6–20.3)
Overweight 25–30 kg/m^2^	542, 796	28.0	(24.1–32.3)	590, 685	15.0	(12.1–18.5)
Obese 30–35 kg/m^2^	217, 326	27.9	(22.0–34.7)	272, 306	19.6	(14.8–25.6)
Obese >35 kg/m^2^	87, 128	44.6	(34.3–55.3)	165, 182	24.3	(17.4–32.9)
**Number of self-reported chronic conditions**						
None	440, 656	14.4	(11.2–18.4)	537, 631	8.4	(6.1–11.5)
One	423, 614	24.2	(20.1–28.7)	556, 638	13.0	(10.1–16.5)
Two or more	420, 587	43.7	(38.7–48.9)	674, 755	27.8	(24.0–32.0)
**Difficulty walking upstairs**						
None	968, 1428	20.1	(17.5–23.0)	1199, 1395	11.5	(9.6–13.7)
Some	215, 294	43.3	(36.1–50.7)	383, 428	23.5	(19.1–28.7)
Much/unable to do	100,135	63.0	52.1–72.7	184,198	42.8	34.7–51.3

### Sexual activity and satisfaction among participants whose health affected their sex lives

Among the sub-group of those participants aged 55–74 years who had a health condition, 62.0% of men and 54.3% of women had been sexually active in the previous six months, and 41.9% of men and 42.1% women reported being satisfied with their sex lives. The proportion reporting recent sexual activity was higher among men and women aged 55–64 years compared with those aged 65–74 years but there were no age-related differences in sexual satisfaction. The proportion reporting recent sexual activity was more than four times as high, and the proportion who were satisfied with their sex lives was nearly twice as high, among those who were cohabiting or in a steady relationship compared with those were not (Tables 3–6).

**Table 3:** Sexual activity in the last 6 months: Men.

*Base*: *Men aged 55–74 with health condition / medication that affects sex life*	% had sex in last 6 months	95% CIs	Adjusted OR^a^	95% CIs	P-value	Base (unwt, wt)
**All men**	62.0	(56.8–67.0)				387,570
**Age group**					0.0003	
-55-64	68.7	(61.6–75.0)	1.00			204, 313
-65-74	53.9	(46.3–61.3)	0.40	0.24–0.66		183, 257
**Relationship status**					<0.0001	
-Living with partner/in steady relationship	70.2	(64.6–75.2)	1.00			297, 383
-Not in steady relationship	14.6	(8.7–23.3)	0.05	0.03–0.11		90, 84
**Economic status**					0.0937	
-Retired	52.9	(45.5–60.2)	1.00			200, 279
-Unemployed	55.9	(43.5–67.7)	0.99	0.44–2.22		72, 105
-Employed	78.9	(70.2–85.5)	1.95	1.00–3.79		114, 185
**Self-reported general health**					<0.0001	
-Very good	80.9	(67.7–89.5)	1.00			47, 71
-Good	72.7	(64.1–79.9)	0.50	0.19–1.35		137, 202
-Fair	56.3	(47.1–65.1)	0.20	0.07–0.54		131, 199
-Bad/very bad	36.7	(25.5–49.6)	0.07	0.02–0.23		71, 96
**Longstanding illness/disability**					0.0028	
-None	77.8	(66.8–85.9)	1.00			84,128
-Not limiting	60.0	(49.7–69.6)	0.39	0.18–0.86		103, 157
- Limiting	56.0	(48.7–63.1)	0.29	0.14–0.59		200, 285
**Body Mass Index (BMI)**					0.0051	
-Underweight/normal	69.5	(59.8–77.8)	1.00			102, 144
-Overweight	65.7	(58.0–72.7)	0.66	0.34–1.27		170, 256
-Obese	51.4	(41.6–61.1)	0.31	0.15–0.65		111, 167
**Difficulty walking upstairs**					0.0013	
-None	69.0	(62.2–75.1)	1.00			225, 334
-Some/much	52.1	(43.9–60.1)	0.49	0.29–0.85		162, 236
**Depressive symptoms**^b^					0.0400	
-No	64.1	(58.5–69.4)	1.00			324, 484
-Yes	49.4	(35.8–63.0)	0.47	0.23–0.97		62, 85
**Taken medication to aid sex (ever)**					0.0115	
-No	57.5	(50.9–63.8)	1.00			254, 381
-Yes	71.2	(61.8–79.0)	2.17	1.19–3.96		133, 189
**Sought professional help regarding sex life (past year)**					0.0204	
-No	57.5	(51.1–63.6)	1.00			273, 407
-Yes	73.3	(64.1–80.9)	1.98	1.11–3.53		114, 164
**Happy with relationship**^c^					0.3396	
-Other	95.4	(86.3–98.5)	1.00			67,112
-Yes	91.0	(85.0–95.4)	0.50	0.12–2.06		119, 194
**Natural to want less sex as get older**					0.1098	
-Disagree/neither/don’t know	68.0	(60.0–75.0)	1.00			155,230
-Agree	58.0	(51.0–64.7)	0.65	0.38–1.10		232, 340
**Easy to talk to regular partner about sex**					0.3562	
Easy	65.5	(58.6–71.7)	1.00			213, 313
Depends/difficult	57.9	(50.1–65.2)	0.78	0.46–1.32		174, 258

^a^Adjusted for age and relationship status (except for ‘age’ row (no adjustment) and ‘relationship status’ row (adjusted for age only)

^b^Measured via validated two-item measure (PHQ-2)

^c^if in steady/cohabiting relationship

**Table 4:** Sexual activity in the last 6 months: Women.

*Base*: *Women aged 55–74 with health condition / medication that affects sex life*	% had sex in last 6 months	95% CIs	Adjusted OR^a^	95% CIs	p-value	Base (unwt, wt)
**All women**	54.3	(48.1–60.4)				281, 366
**Age group**					0.0273	
-55-64	59.5	(51.6–66.9)	1.00			177, 237
-65-74	44.8	(34.5–55.6)	0.52	0.29–0.93		104, 129
**Relationship status**					<0.0001	
-Living with partner/in steady relationship	60.6	(53.9–67.0)	1.00			222, 316
-Not in steady relationship	14.2	(7.1–26.3)	0.10	0.04–0.23		59, 50
**Economic status**					0.0576	
-Retired	47.5	(39.0–56.1)	1.00			146, 182
-Unemployed	57.8	(36.7–63.8)	0.93	0.41–2.15		59, 79
-Employed	70.6	(59.1–80.0)	2.35	1.03–5.38		73, 103
**Self-reported general health**					0.0732	
-Very good	66.8	(50.9–79.6)	1.00			41, 54
-Good	58.9	(47.2–69.7)	0.71	0.28–1.81		81, 108
-Fair	53.2	(43.1–63.1)	0.56	0.22–1.40		96, 119
-Bad/very bad	42.0	(29.5–55.7)	0.31	0.12–0.82		63, 85
**Longstanding illness/disability**					0.1196	
-None	60.7	(47.9–72.2)	1.00			65, 89
-Not limiting	64.0	(43.1–66.0)	1.47	0.56–3.90		49, 64
- Limiting	48.7	(44.2–66.0)	0.66	0.33–1.31		167, 213
**Body Mass Index (BMI)**					0.9609	
-Underweight/normal	52.9	(42.9–62.6)	1.00			101, 135
-Overweight	54.8	(43.1–66.0)	1.02	0.51–2.01		84, 107
-Obese	55.4	(44.2–66.0)	0.92	0.46–1.83		83, 108
**Difficulty walking upstairs**					0.0010	
-None	62.2	(57.4–74.0)	1.00			126, 174
-Some/much	43.6	(35.5–52.1)	0.38	0.22–0.68		155, 192
**Depressive symptoms**^b^					0.0663	
-No	56.6	(49.8–63.2)	1.00			232, 307
-Yes	40.1	(26.2–55.8)	0.50	0.24–1.05		48, 57
**Sought professional help regarding sex life (past year)**					0.0359	
-No	49.6	(42.6–56.5)	1.00			223, 289
-Yes	71.5	(57.6–82.2)	2.33	1.06–5.12		57, 75
**Happy with relationship**^c^					0.6191	
-Other	86.2	(73.4–93.4)	1.00			60, 92
-Yes	91.5	(82.7–96.0)	1.37	0.40–4.74		71, 102
**Natural to want less sex as get older**					0.4639	
-Disagree/neither/don’t know	49.8	(39.5–60.1)	1.00			108, 132
-Agree	56.9	(49.2–64.3)	1.25	0.69–2.29		173, 233
**Easy to talk to regular partner about sex**					0.0013	
Easy	66.3	(57.7–73.9)	1.00			140, 189
Depends/difficult	40.9	(32.5–49.9)	0.39	0.22–0.69		140, 175

^a^Adjusted for age and relationship status (except for ‘age’ row (no adjustment) and ‘relationship status’ row (adjusted for age only)

^b^Measured via validated two-item measure (PHQ-2)

^c^if in steady/cohabiting relationship

**Table 5:** Satisfied with sex life: Men.

*Base*: *Men aged 55–74 with health condition / medication that affects sex life*	% satisfied with sex life	95% CIs	Adjusted OR^a^	95% CIs	p-value	Base (unwt, wt)
**All men**	41.9	(36.6–47.4)				388, 572
**Age group** -55-64	43.3	(36.0–50.9)	1.00		0.4326	204, 313
-65-74	40.3	(33.0–48.0)	0.84	(0.54–1.30)		184, 259
**Relationship status**						
-Living with partner/ in a steady relationship	45.3	(39.3–51.4)	1.00		0.0010	298, 488
-Not in steady relationship	22.2	(14.0–33.4)	0.35	(0.19–0.66)		90, 84
**Economic status**					0.1727	
-Retired	36.6	(29.7–44.0)	1.00			201, 280
-Not working	38.5	(27.0–51.3)	1.36	(0.63–2.93)		72, 105
-Employed	51.4	(41.6–61.0)	1.84	(0.97–3.49)		114, 185
**Self-reported general health**					0.4186	
-Very good	52.4	(37.4–66.9)	1.00			47, 71
-Good	43.3	(34.6–52.4)	0.69	(0.33–1.42)		138, 203
-Fair	40.7	(31.8–50.3)	0.65	(0.31–1.33)		131, 199
-Bad/very bad	34.6	(23.8–47.4)	0.55	(0.24–1.26)		71, 96
**Longstanding illness/disability**					0.7484	
-None	44.8	(33.7–56.4)	1.00			85, 130
-Not limiting	42.1	(32.3–52.5)	0.87	(0.45–1.67)		103, 157
- Limiting	40.5	(33.4–48.1)	0.80	(0.45–1.42)		200, 285
**Body Mass Index (BMI)**					0.2522	
-Underweight/normal	46.2	(36.0–56.8)	1.00			102, 144
-Overweight	43.5	(35.6–51.8)	0.78	(0.44–1.39)		171, 257
-Obese	35.8	(26.7–46.0)	0.58	(0.30–1.11)		111, 167
**Difficulty walking upstairs**					0.0576	
-None	47.2	(40.3–54.2)	1.00			226, 336
-Some/much	34.4	(27.0–42.6)	0.63	(0.39–1.02)		318, 468
**Depressive symptoms**^b^					0.0155	
-No	44.9	(39.0–50.9)	1.00			325, 486
-Yes	24.0	(13.8–38.2)	0.38	(0.17–0.83)		62, 85
**Sexually active *in last 6 months***					<0.0001	
-No	23.6	(17.2–31.4)	1.00			166, 217
-Yes	53.3	(46.2–60.2)	3.17	1.87–5.37		221, 354
**Vaginal intercourse *in last 6 months***					<0.0001	
-No	23.2	(17.4–30.2)	1.00			201, 272
-Yes	59.3	(51.6–66.6)	4.98	(2.99–8.29)		183, 295
**Oral sex *in last 6 months***					0.0017	
-No	33.7	(27.8–40.1)	1.00			257, 368
-Yes	57.6	(48.1–66.5)	2.53	(1.52–4.20)		128, 201
**Genital contact *in last 6 months***					0.0704	
-No	34.1	(27.7–41.2)	1.00			225, 309
-Yes	51.5	(43.0–59.9)	1.75	(1.08–2.85)		159, 257
**Taken medication to assist sexual performance**					0.0944	
-No	44.7	(38.1–51.5)	1.00			255, 382
-Yes	36.3	(28.0–45.5)	0.68	(0.42–1.10)		133, 189
**Sought help regarding sex life (past year)**					0.0549	
-No	44.5	(38.2–51.1)	1.00			274, 408
-Yes	35.3	(26.3–45.5)	0.60	(0.35–1.01)		114, 164
**Happy with relationship**^c^					0.0800	
-Other	48.4	(36.1–60.9)	1.00			67, 112
-Yes	64.3	(54.7–72.9)	1.83	(0.93–3.59)		119, 194
**Natural to want less sex as get older**					0.1863	
-Disagree/neither/don’t know	45.8	(37.3–54.6)	1.00			155, 230
-Agree	39.3	(32.6–46.4)	0.77	(0.48–1.24)		233, 342
**Preferred frequency of sex**					<0.0001	
More often than I do now	39.3	(31.5–47.7)	1.00			153, 248
Right as it is/less often	85.6	(71.3–89.3)	8.44	(3.89–18.33)		81, 123
**Easy to talk to regular partner about sex**					0.0039	
Easy	49.6	(42.5–56.9)	1.00			213, 313
Depends/difficult	32.6	23.4–40.7	0.53	(0.33–0.86)		175, 259

^a^Adjusted for age and relationship status (except for ‘age’ row (no adjustment) and ‘relationship status’ row (adjusted for age only)

^b^Measured via validated two-item measure (PHQ-2)

^c^if in steady/cohabiting relationship

**Table 6:** Satisfied with sex life: Women.

*Base*: *Women aged 55–74 with health condition / medication that affects sex life*	% satisfied with sex life	95% CIs	Adjusted OR^a^	95% CIs	p-value	Base (unwt, wt)
**All women**	42.1	(36.1–48.4)				279, 363
**Age group**					0.7959	
-55-64	41.5	(34.1–49.3)	1.00			176, 235
-65-74	43.2	(33.4–53.6)	1.07	(0.64–1.80)		103, 128
**Relationship status**					0.0214	
-Living with partner/ in a steady relationship	44.5	(37.8–51.5)	1.00			221, 314
-Not in steady relationship	26.3	(16.2–39.7)	0.45	(0.23–0.89)		58, 49
**Economic status**					0.6164	
-Retired	44.5	(36.1–53.3)	1.00			145, 181
-Not working	43.1	(30.9–56.2)	1.27	(0.48–3.35)		58, 77
-Employed	37.5	(26.8–50.6)	0.84	(0.37–1.90)		73, 103
**Self-reported general health**					0.6545	
-Very good	43.7	(27.8–61.1)	1.00			41, 54
-Good	46.5	(35.1–58.3)	1.33	(0.52–3.40)		80, 107
-Fair	35.4	(25.8–46.2)	0.93	(0.36–2.40)		95, 116
-Bad/very bad	44.7	(32.1–57.9)	1.44	(0.55–3.83)		63, 85
**Longstanding illness/disability**					0.4504	
-None	48.7	(35.7–61.9)	1.00			65, 79
-Not limiting	34.8	(22.0–50.2)	0.59	(0.25–1.41)		48, 63
- Limiting	41.5	(33.9–49.5)	0.89	(0.45–0.68)		166, 210
**Body Mass Index (BMI)**					0.7439	
-Underweight/normal	42.1	(31.9–52.9)	1.00			100, 134
-Overweight	37.5	(27.5–48.7)	0.94	(0.48–1.84)		84, 107
-Obese	45.9	(34.6–57.6)	1.22	(0.63–2.38)		82, 106
**Difficulty walking upstairs**					0.7839	
-None	43.4	(34.2–53.1)	1.00			125, 173
-Some/much	40.9	(33.1–49.2)	1.09	(0.63–1.89)		154, 190
**Depressive symptoms**^b^					0.0993	
-No	44.9	(38.1–51.9)	1.00			231, 306
-Yes	26.9	(15.6–42.4)	0.51	(0.23–1.14)		48, 57
**Sexually active *in last 6 months***					0.0009	
-No	29.0	(21.6–37.6)	1.00			138, 166
-Yes	53.2	(44.3–61.8)	2.87	(1.54–5.36)		141.197
**Vaginal intercourse *in last 6 months***					0.0001	
-No	29.5	(22.3–37.8)	1.00			158, 195
-Yes	56.8	(47.3–65.8)	3.77	(1.96–7.26)		121, 168
**Oral sex *in last 6 months***					0.0705	
-No	37.3	(30.5–44.7)	1.00			197, 245
-Yes	51.5	(39.7–63.0)	1.80	(0.95–3.39)		81, 117
**Genital contact *in last 6 months***					0.0323	
-No	35.7	(28.7–43.3)	1.00			180, 223
-Yes	51.9	(41.5–62.1)	1.86	(1.05–3.29)		98, 138
**Sought help regarding sex life (past year)**					0.1353	
-No	43.3	(36.7–50.2)	1.00			223, 289
-Yes	37.3	(24.6–52.0)	0.58	(0.28–1.19)		56, 74
**Happy with relationship**^c^					0.0299	
-Other	39.5	(27.3–53.1)	1.00			60, 92
-Yes	66.6	(54.4–76.9)	2.55	(1.10–5.94)		71, 102
**Natural to want less sex as get older**					0.5972	
-Disagree/neither/don’t know	43.8	(34.1–54.0)	1.00			107, 131
-Agree	41.1	(33.5–49.2)	0.86	(0.48–1.53)		172, 231
**Preferred frequency of sex**					<0.0001	
More often than I do now	33.1	(22.9–45.2)	1.00			81, 110
Right as it is/less often	66.1	(55.0–75.7)	6.38	(2.65–15.40)		88, 129
**Easy to talk to regular partner about sex**					0.0007	
Easy	53.5	(44.8–62.0)	1.00			139, 188
Depends/difficult	29.9	(22.3–38.7)	0.37	0.21–0.66)		140, 175

^a^Adjusted for age and relationship status (except for ‘age’ row (no adjustment) and ‘relationship status’ row (adjusted for age only)

^b^Measured via validated two-item measure (PHQ-2)

^c^if in steady/cohabiting relationship

Among this sub-group, self-reported general health was still strongly associated with recent sexual activity, especially for men. After adjusting for age and relationship status, the odds for being sexually active were much lower for those who saw themselves as being in bad/very bad health compared with those in very good health (with adjusted odds of 0.07 for men and 0.31 for women). For men, but not women, a similar association was found for longstanding illness; the adjusted odds for recent sexual activity for men reporting a limiting longstanding illness was 0.29 relative to those with no longstanding illness.

Individuals were more likely to report recent sexual activity if they reported no mobility difficulties (men and women); normal weight compared with being obese (men only); having no depressive symptoms (men only); or being employed compared with retired.

Sexual activity in the past six months was associated with the use of medication to aid sex (men only); seeking help regarding their sex life (men and women); and finding it easy to talk to their regular partner about sex (women only).

By contrast, after adjustment for age and relationship status, satisfaction with sex life showed no significant association with self-reported general health or with any other physical health variables. Satisfaction was most strongly associated with sexual activity in the past 6 months. Adjusted odds for satisfaction with sex life were much higher among both men and women reporting sexual activity in the past 6 months compared with the sexually inactive (with adjusted odds of 3.17 for men and 2.87 for women). The magnitude of the difference was considerably greater for having vaginal intercourse compared with oral sex or genital stimulation, particularly among men. Men and women were also much more likely to be satisfied with their sex life if they felt that the frequency of sex was about right (with adjusted odds of 8.44 for men and 6.38 for women). Experiencing depressive symptoms was significantly associated with lower odds of sexual satisfaction in men, but not women, and those who found it easy to communicate with a partner about sex were more likely to be satisfied. For women (but not men) in a steady or cohabiting relationship, after adjusting for age, satisfaction with their sex life was associated with feeling happy in the relationship.

### Perceptions of how health conditions affect sexual activity and enjoyment

While the associations between age, health and sexual activity observed in the survey data were also evident in the in-depth accounts, many participants found it difficult to separate the effects of declining health from those of increasing age. Ill health was seen as accelerating an inevitable decline in sexual activity with age which made it easier to accept:

*…*.*as you get older*, *certain things in life compensate for [sex]*. *I’ve got companionship and peace and quiet and a nice comfortable home so I won’t be missing it now*. (M3)

Participants often found it difficult to elaborate on the link between ill health and sexual activity in the in-depth interviews. Establishing cause and effect required them to retrieve information on two aspects of the association: first, specific health conditions to which changes in sexual activity might be attributed, and second, the sequence in which these events had occurred. Both posed challenges. For some, the multiplicity of ailments, and variation in timing of onset and severity, created problems for attribution and recall. Rarely were symptoms of ill-health experienced in isolation from one another, and it was hard for participants to isolate their effect.

*[…] I wouldn’t have thought it’s down to me colitis because I’ve had that for years and it never had any impact*, *you know*. *I mean even after me* [blood pressure] *tablets and if I’m saying well maybes* [the decline in frequency of sex] *started then*, *if it did it was only slightly*, *it wasn’t a big deal*. *But likes of now*, *you know*, *being with the combination of maybe being older*, *my TIA* [transient ischaemic attack] *may have had an effect*. *But you see I don’t know […] I can’t honestly say*, *“Oh yeah*, *it was since then*,*” definitely*, *you know*, *I can’t say that because I think it’s just been gradual*, *you know*. (M8)

With regard to timing, it was not always possible to recall the sequence in which health-related events had occurred, particularly where the onset of ill health had been gradual and symptoms intermittent. Similarly, where the onset of ill health occurred simultaneously with life events such as bereavement (M3), it was difficult to disentangle the influences and assert attribution.

Despite the uncertainties around the order of events and precise causes of sexual difficulties, most participants were able to describe specific ways in which they felt aspects of their health had affected their sex lives. For some, the health condition impacted directly on the capacity to have sex. Nine of the 11 men (M1, M2, M3, M4, M6, M7, M8, M10, M11) and one woman (M13) (in relation to her partner), saw illness or medication as having led to erectile problems, making penetrative intercourse difficult or impossible to achieve. For two women, conditions that caused sex to be painful, such as cystitis (W8) and severe back pain (W2), had a direct bearing on sexual frequency and enjoyment. As conventionally practised, sexual activity requires a degree of agility, and musculo-skeletal deterioration, accidental damage, or the aftermath of surgical procedures were reported in more than one account as restricting mobility (W2, W9).

Medication and procedures aimed at alleviating health conditions were also seen as having had a direct and detrimental effect on sexual enjoyment (M2, W1, W4, W8). Complicated treatment regimens interrupted the spontaneity of sex. One woman described the impact of remedies for her gynaecological problems–a vaginal ring for uterine prolapse and sanitary pads for her weak bladder:

*By the time you put the [vaginal] ring in and […] put your Tena Lady (pad) on and think “God*!*” and then you think about it*, *that would put you off a sex life you must admit* (W5).

For others, health-related factors were less direct. A husband’s sleep apnoea, for example, or excessive fidgeting because of pain occasioned a move into separate beds, and since sex was associated with being in bed, that too ceased along with the move. Changes to sleeping arrangements, initially intended as temporary, sometimes became permanent. A woman who had recently undergone a hysterectomy asked her husband to move to a separate bed because she was afraid he would knock her stitches and he had never moved back in (W1). Fatigue resulting from ill health also had an impact on sexual activity, as in the case of a participant with diabetes and a thyroid problem (W6).

Some participants described their fear of exacerbating an existing health condition. One woman had suffered recurrent bouts of cystitis following a hysterectomy in her early 40s and believed that having sex triggered episodes:

*Yes*, *you do*, *you worry*. *After you’ve had sex*, *you think*, *‘What will it be like in the morning*? *Will I be sore*, *will I be alright*, *will I pick up another dose [of cystitis]*?*’* (W8).

Such fears were more apparent among those who had experienced life-threatening events such as a heart attack or stroke. One man feared that use of PDE-5 inhibitors (such as sildenafil) for his erectile difficulties would prompt another heart attack and, seeing his choice as between living and having no sex life or ‘*dying happy’* (M2), chose the former. Another, who had previously experienced a transient ischaemic attack (or mini-stroke), took a different view: ‘*I can’t be worrying about that all the time; it might never happen*’ (M8).

As the survey data showed, experience of depression was associated with sexual inactivity. In-depth interviews suggested that, in some cases, depression occurred in the absence of other illness (W4; M5), in others it was attributed to physical illness such as myalgic encephalomyelitis (ME) (M1) or Parkinson’s disease (W2; W10), whilst for others it was seen as a consequence of generally worsening physical health (W5). Feeling depressed was described as dampening sexual desire: ‘*the last thing you want to think about’* (W4), and where this impacted detrimentally on the relationship, it in turn worsened mood, setting up a vicious spiral. Accounts of depression also shed light on the higher prevalence of sexual activity among employed as opposed to retired participants. Although one man described retirement having resulted in more time to enjoy sex (M1), others described difficulties adjusting to loss of status and routine (M4) and a concomitant decline in mental health which then impacted negatively on sexual activity and satisfaction.

In asking whether a health condition affected sexual activity and enjoyment, we assumed the effect would have been adverse, but this was not always the case. A woman in her mid-sixties (W10), for example, felt that the medication she was taking for Parkinson’s disease had reawakened an interest in sex that had waned since the menopause, and her relationship with her husband had become closer as a result. In two other cases, erectile difficulties—experienced by a participant in one, and by the participant’s partner in another—had a positive effect on sexual enjoyment by requiring a more imaginative approach to lovemaking, greater variation in sexual repertoire, and sessions of longer duration (M1, W7).

### The relationship context

In the survey data, an important determinant of whether participants were sexually active was whether they were living with a partner or in a steady relationship. Several accounts illustrated how health issues impacted fundamentally on the ability to find a partner. For a 59 year-old widow (W11) with a complex range of chronic health problems, her restricted mobility limited opportunities for getting out and meeting people; and for a man with erectile difficulties resulting from surgery (M10), lack of confidence deterred him from seeking a new relationship. These problems were compounded by lack of motivation, anxieties based on previous experiences and feeling too old to consider starting anew.

For men and women with a steady or cohabiting partner, the survey finding that happiness in the relationship was more strongly related to satisfaction with their sex lives than to sexual activity was reflected in the in-depth accounts. Where the relationship was close, men and women described compensatory mechanisms such as an ability to make light of the problem, finding other sources of intimacy, or adjusting to a decline in frequency of sex. These adjustments may have involved some *ex-post facto* rationalisation, but not having sex seemed to matter less where the relationship was good: ‘*I’d rather him a bit more happy and loving than have the sex’* (W12).

Others, both women (W1, W8), described continuing to have sex despite a health-related decline in their own sexual desire, to please their partner (W1) and to ensure the continued stability of the relationship:

*Well we still dabble but more on his behalf than mine […] I think*, *‘Well*, *it’s not fair for him to have to go without because I had that operation’* (W1).

Where the relationship was less close, health problems either provided an excuse to end sexual activity or else deterred attempts to resolve sexual difficulties. For one woman, the effect of her gynaecological problems on her sex life had been relevant, but the more significant influence was her poor relationship with her husband (W5).

Ill health also affected sexual activity and satisfaction via its impact on the quality of the relationship. Declining health sometimes affected the balance of the relationship by leading to new roles and dependencies. Partners were not always able to adjust to a caring role and, conversely, unhealthy individuals could feel unsupported by their partners (W2, W5) creating rifts in the relationship. Sexual problems triggered by ill health impacted negatively on the relationship, for instance where one partner attributed blame even where ill health was known to be a contributing factor (M4).

Where sexual activity had ceased because of health issues on the part of one or other partner, the loss of physical intimacy had clear consequences for the relationship:

*…I haven’t had sex for 18 months and that’s because my husband’s got very bad arthritis and he takes beta-blockers*, *and because of that he finds it very difficult*. *[*.*…] I really do miss the closeness of the cuddles before and the cuddles after*. *I feel really sorry for my husband*, *I think he misses it more than I do* (W12).

### Responses to the impact of ill health on sexual activity and enjoyment

In the survey, approximately half of the women and a third of the men who reported a health condition affecting sexual activity, had not had sex in the previous six months. In-depth accounts indicated that when penetrative intercourse became difficult or impossible, it generally signalled an end to sexual activity. This was not necessarily problematic. For some women, health problems provided a welcome respite from sex, though this view was not expressed by any of the men we interviewed.

Participants who continued to be sexually active had often made adjustments to the way they had penetrative sex. For the most part, these were limited to changes in positions:

*P*: *We don’t have ….different positions and things like that, it’s just one, and that’s it. I mean, usually he likes to stand up because he’s had two knee operations so he can’t bend down. He can’t kneel on them anymore*.

*I*: *So that affects the position that you can have sex in?*

*P*: *Yeah*, *doggy-style as I put it*. *I know it sounds rude*, *but you understand what I mean* (W1).

More substantive changes to sexual practices were less commonly mentioned. There were isolated instances in which health issues had forced a re-evaluation of what counted as satisfactory sex. An elderly woman with a raft of health complaints including arthritis and emphysema, and her non-live in partner who had bowel cancer and heart disease, had devised an imaginative routine for the occasions on which, as she said, ‘*it doesn’ae go up’* (W7). Their ‘*half hour on the bed every afternoon*’ meant enjoying kissing, cuddling and petting. In the case of others, the need to find alternatives to their previous repertoires had actually improved their enjoyment. The sexual relationship of a 75 year-old man with a 70 year-old partner which, prior to his illness had been reportedly unsatisfactory, was now flourishing as they had been forced to explore alternatives to penetrative sex as a result of his erectile difficulties (M1).

For most participants, though, sex was equated with penetration, and the idea of pursuing alternatives had little appeal. Generally, this seemed to be a matter of habit and familiarity. Men and women–some ruefully–claimed it was ‘too late in the day’ to broaden their repertoire (M3). Over the years, they had adopted practices they found enjoyable and, if these were no longer possible, they had no appetite for substitutes: ‘*you get used to what you like and because I can’t have what I like I’m not really bothered’* (W12). One man had bought a vibrator on a weekend away but it had proved unsatisfactory. The accounts of others, however, revealed a more profound distaste for non-penetrative sex:

*P*: *I still cuddle her, we still caress one another occasionally, but no, I think intercourse is important*.

*I: So that’s kind of what sex is for you*.

*P*: *Oh yes*, *I think so*, *yeah*, *absolutely*, *I would never dabble with anything but intercourse really*, *we’ve never gone into oral sex or anything of that nature*. *Obviously*, *there’s foreplay*, *etc*, *which every couple*, *I hope*, *indulge in*, *but an actual sex act itself has got to be full intercourse as far as I’m concerned* (M3).

### Help seeking

Among survey participants aged 55–74 years with a health condition affecting their sex life, about one in four had sought help or advice about their sex life in the past year (28.6% of men and 20.5% of women. One in three (33.1%) men reported taking medication in the past year to enhance sexual performance (e.g. sildenafil). Asked why they had not sought medical advice in the in-depth interviews, several participants said they did not see this as a priority for their health practitioner. One man felt he needed to first tackle his obesity before seeking help for his sexual problems, another felt he had caused his circulatory problems himself through smoking, and another felt he was making enough demands on his doctor’s time already:

*… since I’ve had me heart trouble*, *I’ve said to meself*, *you know*, *season ticket in and out ‘t doctor’s*. *‘Oh*, *bloody hell*! *Not him again*!*’ you know*. *This is what it feels like* (M2).

In two cases, however, it was through the act of seeking help for sexual problems that the underlying health problem had been discovered.

Where help had been received, it was generally in the form of sildenafil (‘*little blue pills’*) for men. No participants in the qualitative component mentioned any other form of help. The survey data showed that the proportion taking medication in the last year to enhance sexual performance (e.g. sildenafil) (one in three) was higher than the proportion (about one in four) reporting having sought help from health care professionals, suggesting alternative sources of supply. Use of sildenafil was not significantly associated with sexual satisfaction in the survey data and this was confirmed in the in-depth interviews. Several men described having used sildenafil, but with varying degrees of success (M4) and with varying degrees of satisfaction (M9; W1).

## Discussion and implications

Our survey data suggest that a sizeable number of older people in Britain—more than one in four men and one in six women—see themselves as having a health condition affecting their sexual activity or enjoyment. Of those, over a third of men and almost half of women have not been sexually active in the past six months, and only two in five men and women say they are satisfied with their sex lives.

The qualitative data show a range of ways in which health can impact on sexual activity. Most fundamentally, ill health may influence whether individuals have a partner with whom to have sex. Health conditions also operate through the inclination and capacity to be sexual, the practical possibilities for doing so and the limits placed on forms of sexual expression. The effect of declining health on sexual activity goes hand in hand with that of advancing age, making it difficult to determine which is the stronger influence.

Whether men and women are satisfied with their sex lives depends, most notably, on whether they are having sex at all, as well as on the kind of sex they are having, vaginal intercourse seeming to outweigh other practices in terms of inducing satisfaction. Only a minority seek professional help, and the desire or ability to adapt or modify sexual routines and practices does not appear to be widespread. Satisfaction with their sex lives is also strongly associated with the quality of communication with their partner and, for those with a regular partner, contentment with their relationship. In-depth accounts show this association to be bi-directional: sexual problems may impact negatively on the quality of relationships and vice versa.

An important strength of this study lies in the combination of data from a population-based survey alongside detailed accounts of selected survey participants. Our qualitative work facilitated understanding of mechanisms at work in the association between health status, relationships, sexual activity and satisfaction. It also allowed us to identify explanatory factors which were not measured in the survey, or were measured but not initially entered into the analysis. This mixed-method approach has rarely been taken with respect to older people’s sexuality.

Obtaining men and women’s own explanations of how ill health affects their sex lives also lessens the risk of over-interpreting associations revealed in the quantitative data. The qualitative research has highlighted that the statistical association between poorer health status and less frequent sexual activity tends to simplify complex accounts. Although men and women may have a health condition which affects sexual activity and enjoyment, explanations may be found in pre-existing or co-existing factors, the quality of their relationship, or other life events.

In terms of sampling our study has both strengths and limitations. The data come from a nationally representative survey, which also provided a sampling frame for the qualitative sample. However, Natsal-3 had an upper age limit of 74 years, and so we are unable to describe the experience of people at older ages. In addition, Natsal-3 sampled only people living at private residential addresses, excluding residents of care homes (although the proportion of 55–74 year olds in England and Wales resident in a care home was less than 1% in the 2011 population census). Since both these groups are likely to be in poorer health, our findings may be less generalizable to individuals with more severe health problems.

It is common when identifying weaknesses of studies to point to problems of recall. Yet in the current context–that of informing efforts to help men and women to achieve sex lives that best suit them–difficulties in remembering sequences of life events, illness episodes, and the ups and downs of relationships, are important checks on an over-reliance on memory and can be seen as useful data in their own right.

Our results have implications for public health policy and practice. The finding that the majority of older men and women do not seek help for their sexual difficulties has been shown by others [14, 18, 28]. Health care professionals have been urged to be more proactive in helping their older patients to achieve a satisfying sex life (5, 18). However, our data prompt caution in assuming universality of the need to provide such advice. Some older people are more accepting of not having a sex life than others. Such caution does not lessen the importance of sensitive enquiries about an older person’s sexual function and activity, however, as not to do so may not only be detrimental to the well-being of patients [4, 8, 14] but, as others have observed and our data support, it might lead to underlying medical conditions being inadvertently missed [28].

What seems clear is the need for a holistic approach when dealing with sexual problems in older people, taking account of lifestyle factors, needs and preferences in relation to sexual activity. Our finding, and that of other studies, that declining health may be only one of many factors influencing sexual activity and satisfaction in later life [1, 16] is important in the context of counselling. Most evidently in this context, attention needs to be given to relationship factors [5] and care is needed not to make assumptions that patients have a partner.

Given the multiplicity of factors influencing their sexual activity and satisfaction, it seems especially important not to over-medicalise treatment options for older men and women. Both our survey and qualitative findings suggest that largely pharmacological remedies offered to older people do not necessarily increase sexual satisfaction.

As to what form counselling might take, our data suggest that advice on alternatives to penetrative sex, seen by many as important [4, 13, 17, 20, 28], needs to be delivered with care and tact. Although a minority of participants found alternative ways of expressing their sexuality in response to health-related difficulties and reported positive consequences for sexual satisfaction as a result, the evidence is that the orthodoxy of the equation between sex and penetration is strongly held among older people. For men and women in longer relationships, established patterns of sexual expression are likely to have been influenced by social norms operating in their youth, when alternative practices to vaginal sex were less prevalent. Clearly many find it hard to vary their sexual repertoires later in life. Some may welcome being given ‘permission’ to adopt different practices, but for others such advice may be unpalatable, and tact is needed to determine which applies in individual cases.

Our finding that sexual activity often ceases following major illness is consistent with that of other research [14, 29].The evidence is that the increased risk of coronary heart disease associated with sexual activity is limited and cardiovascular medicine is only weakly associated with erectile dysfunction [29]. More effective advice after diagnosis might reverse the reduction in sexual activity and lead to improved quality of life.

As others have commented, despite the importance of sexual activity and satisfaction to well-being and quality of life, this aspect of older people’s health has received inadequate public health attention [14, 30]. Our hope is that these data, and those from similar studies, may contribute to the evidence base for this to be remedied.
